# A Plasmonic Biosensor Based on Light-Diffusing Fibers Functionalized with Molecularly Imprinted Nanoparticles for Ultralow Sensing of Proteins

**DOI:** 10.3390/nano12091400

**Published:** 2022-04-19

**Authors:** Francesco Arcadio, Mimimorena Seggio, Domenico Del Prete, Gionatan Buonanno, João Mendes, Luís C. C. Coelho, Pedro A. S. Jorge, Luigi Zeni, Alessandra Maria Bossi, Nunzio Cennamo

**Affiliations:** 1 Department of Engineering, University of Campania Luigi Vanvitelli, Via Roma 29, 81031 Aversa, Italy; francesco.arcadio@unicampania.it (F.A.); domenico.delprete@unicampania.it (D.D.P.); gionatan.buonanno@gmail.com (G.B.); luigi.zeni@unicampania.it (L.Z.); 2 Department of Biotechnology, University of Verona, Strada Le Grazie 15, 37134 Verona, Italy; mimimorena.seggio@univr.it (M.S.); alessandramaria.bossi@univr.it (A.M.B.); 3 INESC TEC–Institute for Systems and Computer Engineering, Technology and Science, Faculty of Sciences, University of Porto, 4169-007 Porto, Portugal; joaomendes.quimica@gmail.com (J.M.); luis.c.coelho@inesctec.pt (L.C.C.C.); pedro.jorge@fc.up.pt (P.A.S.J.); 4 Departmento de Física e Astronomia, Faculdade de Ciencias, Universidade do Porto, Rua do Campo Alegre, s/n, 4169-007 Porto, Portugal

**Keywords:** light-diffusing fibers (LDFs), surface plasmon resonance (SPR), molecularly imprinted polymers (MIPs), molecularly imprinted nanoparticles (nanoMIPs), biosensors, plasmonic optical fiber biosensors, specialty optical fibers

## Abstract

Plasmonic bio/chemical sensing based on optical fibers combined with molecularly imprinted nanoparticles (nanoMIPs), which are polymeric receptors prepared by a template-assisted synthesis, has been demonstrated as a powerful method to attain ultra-low detection limits, particularly when exploiting soft nanoMIPs, which are known to deform upon analyte binding. This work presents the development of a surface plasmon resonance (SPR) sensor in silica light-diffusing fibers (LDFs) functionalized with a specific nanoMIP receptor, entailed for the recognition of the protein human serum transferrin (HTR). Despite their great versatility, to date only SPR-LFDs functionalized with antibodies have been reported. Here, the innovative combination of an SPR-LFD platform and nanoMIPs led to the development of a sensor with an ultra-low limit of detection (LOD), equal to about 4 fM, and selective for its target analyte HTR. It is worth noting that the SPR-LDF-nanoMIP sensor was mounted within a specially designed 3D-printed holder yielding a measurement cell suitable for a rapid and reliable setup, and easy for the scaling up of the measurements. Moreover, the fabrication process to realize the SPR platform is minimal, requiring only a metal deposition step.

## 1. Introduction

Surface plasmon resonance (SPR) is a widely adopted principle for the detection of small refractive index variations at the interface between a thin metal nano-film (e.g., gold, silver, etc.) and a dielectric medium (e.g., liquid samples containing analytes that interact with a monolayer of receptors fixed on the SPR surface). By changing the receptor in contact with the metal nano-film, the same SPR transducer can be used to detect different substances [1]. Given this versatility, SPR has been successfully implemented as a sensing mechanism in very diverse fields of application, ranging from environmental monitoring to medical and biochemical applications [2,3,4,5].

The SPR sensor’s landscape relies on a variety of optical configurations, from the prism-based Kretschmann configuration to optical fibers and planar waveguides [6,7,8,9]. In particular, the use of optical fibers and waveguides leads to many advantages mainly related to low cost, small size, and the possibility of remote interrogation, in the context of smart sensor systems. Plasmonic optical fiber platforms have been effectively coupled to several kinds of selective receptors specific to a given target substance, or to a class of substances. Both biological receptors, such as antibodies, enzymes, and aptamers, and synthetic receptors, such as molecularly imprinted polymers (MIPs), have been exploited to functionalize the sensors, yielding ground-breaking bio/chemical sensors [10,11,12,13,14]. 

MIPs are tailor-made receptors, prepared by a template-assisted synthesis [15]. The use of MIPs in optical sensing leads to several advantages, mainly: the possibility to tune the selectivity towards any target analyte, from small molecules to proteins [13,16,17,18]; the possibility to customize any optical configuration with MIPs; MIPs’ synthesis is cheap; MIPs tolerate solvents and temperature extremes, when compared with biological receptors; and they are re-usable. Moreover, MIPs can be synthesized in the form of nanoparticles, called nanoMIPs, or referred to as plastic antibodies [19]. These nanoMIPs can be prepared in sizes ranging from 10 nm to about 200 nm, bearing a limited number of binding sites per nanoparticle; moreover, being nanoparticles, they are characterized by a high surface to volume ratio, enabling fast mass transfer kinetics [20,21]. Additionally, nanoMIPs can be entailed of responsive properties [22]. It has been demonstrated that when SPR plasmonic sensors are functionalized with such soft nanoMIPs, the binding events produce a high variation of the refractive index when it occurs, hence the sensor’s performance is improved [23,24].

Recently, among the class of plasmonic optical-fiber-based sensors, those based on light-diffusing fibers (LDFs) have emerged [25,26,27], together with the plasmonic sensor configurations based on photonic crystal fibers [13,28,29]. More generally, highly sensitive plasmonic-based sensor configurations have been theoretically analyzed and presented in recent years by Deng et al. [30,31]. More specifically for this work, the LDFs can be made either of silica [25,26] or plastic [27] and are characterized by an isotropic light dispersion at the surface, thanks to diverse scattering centers present into the core of the fiber. Because of their particular structures, LDFs constitute an efficient solution for developing plasmonic sensors, since the SPR condition can be easily satisfied. Additionally, the fabrication process is minimal, requiring only a metal deposition to build the SPR sensor [25,26,27]. For this reason, an LDF-based SPR platform was chosen to develop the proposed sensor for ultralow sensing of proteins. Along this line, Cennamo et al. in [32] recently coupled a silica-LDF-based SPR sensor to a bioreceptor to efficiently monitor different concentrations of C-reactive protein (CRP) in human serum. The results obtained showed an improvement in the limit of detection (LOD) of about three orders of magnitude, with respect to a similar plasmonic biosensor based on a plastic optical fiber (POF), functionalized with the same receptor [32].

In this work, for the first time, an SPR-LDF biosensor based on nanoMIP receptors was proposed. The developed sensor exploits the advantages of the LDF platform and the sensitivity gain of nanoMIPs, in order to improve the overall biosensing performances. For this reason, as a proof of concept, the LDF’s gold-covered surface was functionalized with soft nanoMIPs for the specific detection of human serum transferrin (HTR), which is a biomarker of iron metabolism. At the same time, the analytical selectivity of the proposed SPR-LDF-nanoMIP sensor was tested, investigating the responses in the presence of interferent proteins.

## 2. Materials and Methods

### 2.1. Chemicals

The acrylamide (Aam), (R)-α-lipoic acid, 1-ethyl-3-(-3-dimethylaminopropyl) carbodiimide hydrochloride (EDC), N-hydroxysuccinimide (NHS), Lysil-lysine (Lys-Lys), methacrylic acid (MAA), N-tert-butylacrylamide (TBAm), N,N′ -methylene bisacrylamide (BIS), N,N,N′,N′-tetramethyl ethylenediamine (TEMED), ammonium persulfate (APS), sodium dodecyl sulfate (SDS), N-Cyclohexyl-2-aminoethanesulfonic acid (CHES), Tris (hydroxymethyl)-aminomethane (TRIS), sodium dihygrogen phosphate, disodium mono-hydrogen phosphate, hydrochloric acid, sodium hydroxide, sodium chloride, Tween-20, acetonitrile, acetic acid, and ethanol were from Sigma-Aldrich (Darmstadt, Germany). The proteins (human serum transferrin (HTR), pepsin, and horseradish peroxidase) were from Sigma-Aldrich (Darmstadt, Germany).

### 2.2. SPR-LDF Sensor System: Fabrication Steps and Experimental Setup

The presented SPR-LDF sensor was obtained by removing the cladding using a mechanical stripper for a length of 2 cm and, after this, a gold film with a thickness of 60 nm was deposited on the exposed core of the LDF by a sputtering machine (Safematic CCU-010, Zizers, Switzerland). The sputtering process was repeated two times, rotating the fiber by 180°, in such a way that the whole circumference was covered by the gold layer [20].

In this work, a custom holder was designed and printed by a 3D-printer (Photon Mono X UV Resin SLA 3D Printer, Anycubic^®^, Shenzhen, China) in order to fix the SPR-LDF sensor during the tests and to realize a measurement cell. The holder was composed of four clamps used to keep the fiber steady, and it also included a cell to contain the aqueous solution under test. In more detail, the cell dimensions were projected in such a way that the sensible region of the plasmonic sensor was totally immersed in the liquid solution under test.

**Figure 1:**
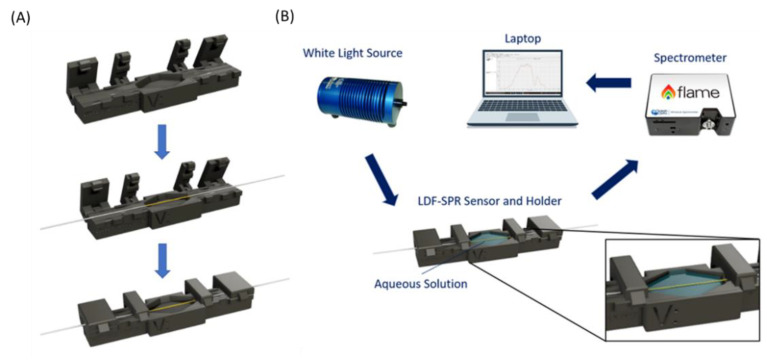
(**A**) The layout of the custom-designed holder used to fix the LDF-SPR sensor and realize the measurement cell. (**B**) The outline of the experimental setup.

Figure 1A shows the layout of the described holder with the steps to fix the SPR-LDF sensor into it. The holder presents the following dimensions: 10 cm in length, 1 cm in width, and 7 mm in thickness. The measurement cell is 2 cm in length, with a depth of about 2 mm and a maximum width of about 1 cm.

To test the proposed plasmonic biosensor, a simple and low-cost experimental setup, illustrated in Figure 1B, was taken into account. Figure 1B also shows a zoom of the measurement cell. More specifically, it consists of a white light source (HL–2000–LL, manufactured by Ocean Optics, Orlando, FL, USA) characterized by a wavelength emission range from 360 nm to 1700 nm and a spectrometer (FLAME-S-VIS-NIR-ES, Ocean Optics, Orlando, FL, USA) with a detection range of between 350 nm and 1000 nm [20,21,22,23]. The SPR-LDF sensor, contained in the 3D-printed holder, was placed in the optical path between the white light source and the spectrometer, and SMA connectors were used to connect all components of the described setup. Finally, the spectrometer was connected to a laptop in order to acquire and process the experimental data.

### 2.3. NanoMIP Synthesis and LDF-SPR Functionalization Process

The nanoMIP synthesis was performed according to [23]. Briefly, a total monomer concentration of 0.1% (*w*/*v*) was used in the syntheses. Acrylamide (Aam), methacrylic acid (MAA), and N-t-butylacrylamide (TBAm) were added at 8, 8, and 4% (mol/mol), respectively, together with 80% (mol/mol) of N,N′-methylenebisacrylamide (BIS) in 20 mM phosphate buffer (PB) with pH 7.4 supplemented with SDS 0.02% (*w*/*v*). The template (HTR) was added to the MIP-vials to the final concentration of 1.2 μM. Vials were closed with rubber caps, sonicated for 10 min, and bubbled with N_2_ for 30 min. Then APS (0.04% *w*/*v*) and TEMED (0.03% *w*/*v*) were added and the polymerization was carried out at 20 °C for 20 h. Control, non-imprinted (nanoNIP) nanoparticles were synthetized using the same protocol but in the absence of the template. At the end of the polymerization the pH was adjusted to 8 with 50 mM Trizma-base, Trypsin was added to the solutions in a 1:25 (*w*/*w*) ratio with respect to the template, and incubated for 2 h at 30 °C. The nanoparticles were then washed with 3 L of MilliQ water using a Vivaflow 50 system (100,000 MWCO) (Sartorius Stedim, Firenze, Italy). The yield of polymerization was calculated from the weight of the lyophilized nanoMIPs with respect to the total weight of the monomers added to the synthetic batch.

SPR-LDF platforms were functionalized with nanoMIPs according to the protocol reported in [23]. Briefly, a self-assembled monolayer (SAM) of carboxylic acids on the LDF was formed by overnight derivatization with 300 μM α-lipoic acid in ethanol 8% v/v. After rising with water, the LDF was incubated for 1 h in 10 mM Lys-Lys, and 50 mM EDC/NHS (1:1 mol:mol) in MES buffer 10 mM pH 5.5. NanoMIPs (10 μM), re-suspended in MES buffer, were added to 50 mM EDC and 50 mM NHS to a final volume of 0.6 mL. An aliquot of 200 μL was placed onto the LDF and allowed to react for 2 h at room temperature in a sealed humid box. NanoMIP-LDFs were then washed extensively in 15 mL Falcon tubes (water 1 h; CHES buffer 10 mM pH 9.3 1 h, water 2 h) prior to use.

## 3. Results and Discussion

To develop the sensor specific for the protein HTR, a receptor layer was added to the plasmonic sensing surface. For that, nanoMIPs tuned for the recognition of HTR were coupled chemically to the gold surface of the SPR platform, with a functionalization pro-cess. This step produces a self-assembled monolayer of nanoMIP, specific for the analyte (the imprinted protein), optically characterized.

**Figure 2:**
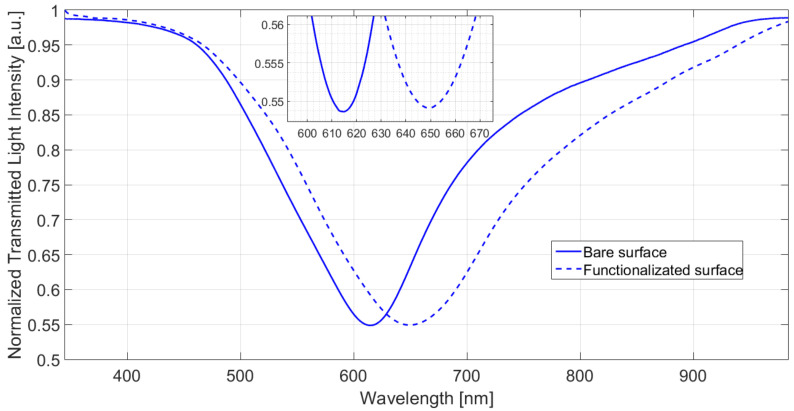
Normalized transmission spectra acquired using water as bulk, before (solid line) and after (dashed line) the nanoMIP’s functionalization.

Surface functionalization was monitored by the optical information of the SPR spectra, as reported in [11]. Figure 2 shows a comparison between the normalized transmission spectra, acquired with water as a surrounding medium, before and after the nanoMIP layer functionalization. The SPR spectra were obtained by normalizing the transmission spectrum in water to the one acquired in air, where the resonance condition is not satisfied (reference spectrum). As shown in Figure 2, for the same bulk medium (water), a resonance wavelength variation toward higher values (red shift) can be noticed before and after the nanoMIPs’ functionalization. Such a red shift, that is, of about 35 nm, confirms the presence of the receptor on the SPR-LDF-sensible surface. Therefore, the effectiveness of the functionalization process was confirmed.

Next, to characterize the SPR-LDF-nanoMIP biosensor functional performance, the HTR-binding response of the sensor was tested with different protein concentrations. Specifically, the ultralow concentrations of HTR were explored, namely the femtomolar range. Indeed, the possibility to measure femtomolar concentrations of the protein-analyte on the sensing platform was demonstrated in earlier works by our group [23,24]. Such ultralow sensitivity was ascribed to the typical deformation of the soft nanoMIPs’ polymer-network structure at binding [33], when the nanoMIPs are closely coupled to the plasmon. Here, we attempted the detection of ultralow HTR concentrations by taking advantage of the combination of soft nanoMIPs with LDF.

**Figure 3:**
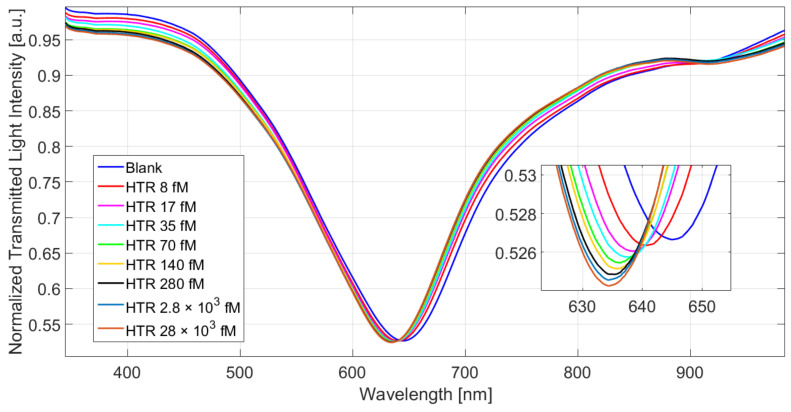
Normalized transmission spectra obtained for different values of HTR concentration (from 8 to 28 × 10^3^ fM). Measurements were performed in triplicate.

Figure 3 shows the SPR spectra for HTR concentration values ranging from 8 to 28 × 10^3^ fM. It can be observed that, when the nanoMIPs bind the analyte, the refractive index of the nanoMIPs greatly decreases and the resonance wavelength shifts to the left (blue shift) [23,24]. The increment in the HTR concentration causes the resonance wavelength’s decrement (blue shift). Such an aspect is supported by the nanoMIPs’ deformation upon the binding event and can be explained as swelling of the nanoMIPs at binding, in coherence with previous studies [23,33]. Once more, as shown in Figure 3, the deformation of the nanoMIPs leads to sensitivity gain, proving a shift of about 6 nm for the HTR concentrations spanning the range from 8 fM to 280 fM, thus proving the exceptional sensitivity of the nanoMIP-enabled plasmonic sensing [23,24].

**Figure 4:**
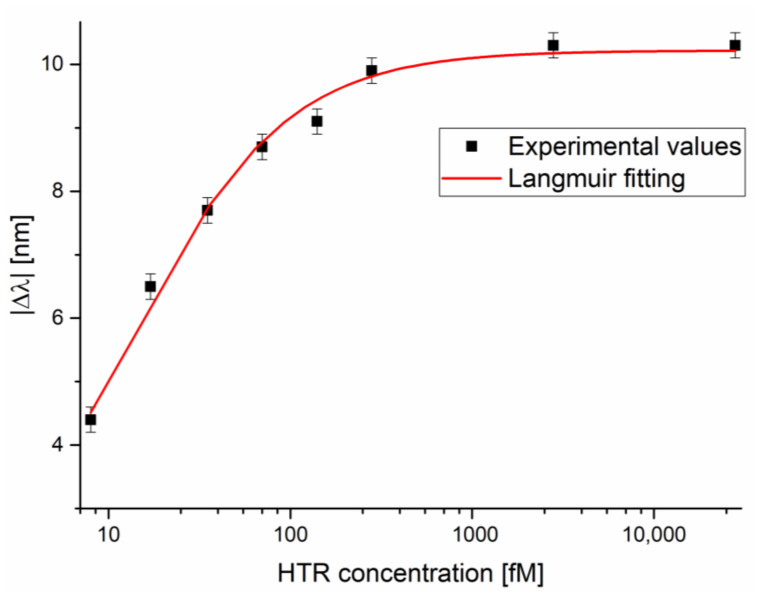
Absolute value of resonance wavelength variation (with respect to the blank) versus the HTR concentration, with the Langmuir fitting of the experimental values in a semi-log scale. Measurements were performed in triplicate.

Figure 4 reports the binding isotherm of HTR on an SPR-LDF-nanoMIP sensor, as the absolute values of the resonance wavelength variation (Δλ), calculated with respect to blank (the solution without analyte), plotted versus the semi-log of the HTR concentration. Measures were performed in triplicate; error bars are reported and 0.2 nm represents the maximum measured variation of the resonance wavelength.

The experimental values shown in Figure 4 were fitted with the Langmuir model equation.

The equation of the fitting curve reported in Figure 4 is reported below:

(1) $$\left|\Delta \lambda \right|=\left|\lambda_{c}-\lambda_{0}\right|=\left|{\Delta \lambda }_{\max}\right|\cdot \left(\frac{c}{K+c}\right)$$

where λ_c_ is the resonance wavelength at the analyte concentration c, λ_0_ is the resonance wavelength value at zero concentration (blank), Δλ_max_ is the maximum value of Δλ (calculated by the saturation value minus the blank value) and K is a dissociation constant.

From Equation (1), when c is much lower than K, i.e., at low analyte concentration, Equation (1) can be considered linear, and the slope is the sensitivity at low concentration. Therefore, the sensitivity at low concentrations can be approximated as:

(2) $$\mathrm{Sensitivity}\;\mathrm{at}\;\mathrm{low}\;c=\frac{{\Delta \lambda }_{\max}}{K}$$

The limit of detection (LOD) can be calculated as the ratio between three times the standard deviation of the blank and the sensitivity at low concentration, as the following is reported:

(3) $$\mathrm{LOD}=\frac{3\times \mathrm{standard}\;\mathrm{deviation}\;\mathrm{of}\;\mathrm{blank}\;\left({\Delta \lambda }_{0}\right)}{\mathrm{Sensitivity}\;\mathrm{at}\;\mathrm{low}\;c}$$

**Figure 5:**
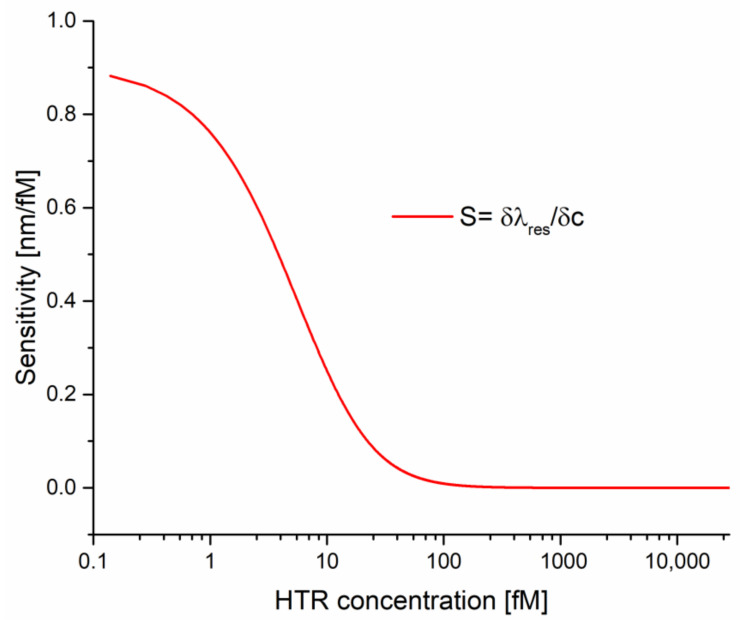
Sensitivity as a function of analyte concentration in semi-log scale.

In Table 1, parameters relative to the Langmuir fitting of the SPR-LDF-nanoMIP shown in Figure 4 are reported. These values were obtained by OriginPro software (Origin Lab. Corp., Northampton, MA, USA). Figure 4 and Equation (1) show that the sensor’s response is non-linear (Langmuir equation). In other words, the sensitivity of the proposed sensor is a function of the HTR concentration. Figure 5 shows the sensor sensitivity curve (sensitivity versus HTR concentration) in a semi-log scale. In this figure, the sensitivity (S) has been calculated as:

(4) $$S=\frac{\partial \lambda_{\mathrm{res}}}{\partial c}$$

In other words, as reported in Equation (4), the sensitivity can be defined by calculating the resonance wavelength shift per unit change in analyte concentration (nm/fM).

The estimated variables reported in Table 1 were used to calculate the biosensor’s parameters that characterize the proposed HTR sensor: affinity constant, sensitivity at low concentration, and detection limit. These values are reported in Table 2, where Equations (2) and (3) were used to obtain them.

**Figure 6:**
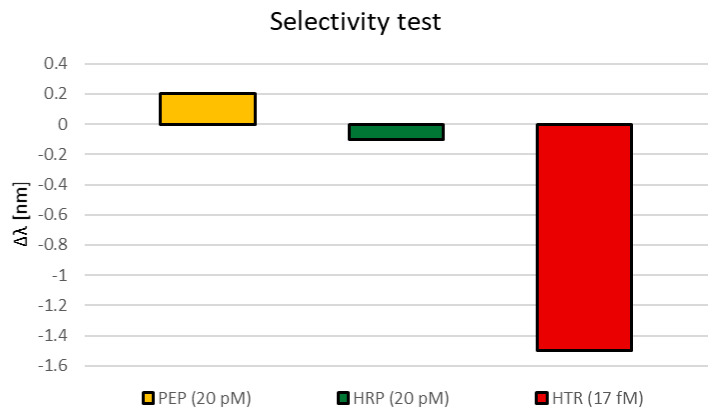
Resonance wavelength variation with respect to blank for different substances.

Finally, a selectivity test was carried out to confirm that the variation in resonance wavelength observed in Figure 3 is due to specific binding events. The selectivity test was performed by considering two different protein interferents, namely pepsin (PEP) and horseradish peroxidase (HRP), at a concentration equal to 20 pM. As shown in the histogram chart in Figure 6, both PEP and HRP were able to produce just slight resonance wavelength variations, equal to 0.2 nm and 0.1 nm, respectively. It is interesting to note the different responses of the proposed SPR-LDF sensor with respect to the two interferents. In fact, the produced shift follows different directions, when considering PEP and HRP, despite both being close to the value of the error bar (0.2 nm). In more detail, the PEP interferent produced a small red shift of the resonance wavelength, attributed to an aspecific deposition of the substance upon the sensitive surface; conversely, HRP produced a blue shift, which is possibly due to a slight interaction with the receptor sites. In any case, the obtained shift values for both interferents can be considered negligible if compared with the shifts produced by the interaction of the nanoMIPs to the analyte (HTR). Indeed, the lowest HTR concentration, equal to 17 fM, and therefore about three orders of magnitude lower than the tested interferents concentrations, produced a blue shift of about 1.5 nm.

To compare the performances of the proposed SPR-LDF-nanoMIP biosensor with the typical responses of other SPR biosensors, Table 3 reports some examples. More specifically, with regards to the LOD values, Table 3 shows a comparative analysis relative to a commercial SPR device (BioNavis Technology Ltd., Tampere, Finland) and different plasmonic sensor configurations developed by our research group and functionalized with different kinds of receptors for substances with molecular weights similar to the HTR analyte herein used.

As shown in Table 3, the works [23,24] present similar LODs to the proposed sensor (femtomolar range), but they are based on SPR probes realized via a difficult manufacturing process. On the contrary, the proposed sensor is based on an SPR probe with a minimal fabrication procedure that requires only a metal deposition step.

## 4. Conclusions

In this work, we reported for the first time on the functionalization of a plasmonic transducer based on light-diffusing fibers (LDF) with a nanoMIP receptor, the latter imprinted for the specific recognition of the protein HTR. The proposed biosensor experimentally tested showed an extreme sensitivity for the detection of ultralow concentrations of analyte, which was set in the low femtomolar range. These results were similar to another plasmonic sensor based on a D-shaped plastic optical fibre (POF) and functionalized with the same soft-nanoMIP receptor. However, compared with the latter, the main advantage of the SPR-LDF-nanoMIP is the simpler fabrication process, since the sensor is prepared just by the deposition of a gold nanolayer on the LDF, opening the possibility to scale up the fabrication of these SPR-LDF-nanoMIP sensors to any analyte. Finally, the SPR-LDF-nanoMIP biosensor set-up was completed by a 3D-printed custom-designed holder that was intended to fix the SPR-LDF sensor and thus to realize the measurement cell, in which the sample can be placed in appropriate volumes, avoiding evaporation issues and thus providing a very stable, robust, and reproducible measurement system.

## Figures and Tables

**Table 1:** Langmuir parameters of HTR detection in buffer solution.

Sensor	$\Delta \lambda_{0}\;[\mathrm{nm}]$		$\Delta \lambda_{max}\;[\mathrm{nm}]$		K [fM]		Statistics	
NanoMIPs combined with SPR-LDF sensor	Value	Standard error	Value	Standard error	Value	Standard error	Reduced Chi-Sqr	Adj. R-square
	0.97	1.03	10.22	0.13	12.87	3.05	1.19	0.99

**Table 2:** Sensor’s chemical parameters for HTR detection in buffer solution.

Sensor	Parameters	Value
SPR-LDF sensor combined with NanoMIPs	K_aff_ [fM^−1^] (K_aff_ = 1/K)	0.08
	Sensitivity at low c [nm/fM]	0.72
	LOD [fM]	4.3

**Table 3:** Comparative analysis between plasmonic biosensors of interest.

Plasmonic Probe	Receptor	Analyte	LOD	Reference
D-shaped plastic optical fiber	nanoMIPs	HTR	1.2 fM	[23]
Nanograting-based chip	nanoMIPs	Bovine serum albumin (BSA)	3 fM	[24]
SPR-LDF sensor	antibody	C-reactive protein	130 fM	[32]
SPR-sensor chip	boronic acid	HTR	4.4 nM	[34]
SPR-LDF sensor	nanoMIPs	HTR	4.3 fM	This work

## Data Availability

The data are available on reasonable request from the corresponding author.
